# Determining Carrageenan Sulfate Groups Using Ion Association with Alcian Blue Dye

**DOI:** 10.3390/molecules31183196

**Published:** 2026-09-10

**Authors:** Alexander Shyichuk, Dorota Ziółkowska, Iryna Schyychuk

**Affiliations:** Faculty of Chemical Technology and Engineering, Bydgoszcz University of Science and Technology, Seminaryjna 3, 85-326 Bydgoszcz, Poland; dorota_z@pbs.edu.pl (D.Z.); iryna.shyychuk@pbs.edu.pl (I.S.)

**Keywords:** carrageenan, Alcian Blue, ion association, sulfate ester content

## Abstract

The purpose of this study is to develop a simple and reliable method to measure sulfate content and determine the purity of carrageenan raw materials. The method uses cationic Alcian Blue dye that binds strongly to anionic carrageenan macromolecules, resulting in an insoluble ion associate. At a specific carrageenan-to-dye ratio, the hydrophobic ion-associated particles form macroscopic flocs and precipitate quickly. The resulting deep sedimentation leaves almost colorless supernatants, indicating the stoichiometric charge ratio. The critical polymer-to-dye ratio has been found to be independent of the dye concentration. The Alcian Blue reagent has been standardized against a synthetic polymer, poly(sodium styrene sulfonate). The method of ion-associate precipitation was used to determine the content of sulfate groups in commercial carrageenans of different types: kappa, iota, and lambda. The results obtained agree well with the IR spectra of the tested carrageenans.

## 1. Introduction

Carrageenan is a highly sulfated polygalactan obtained from red marine algae that has many applications in the food, biomedical, and cosmetic industries [1,2]. The main types of carrageenan are kappa- (κ), iota- (ι), and lambda- (λ) carrageenans, differing in their structural units and sulfation pattern. The repeating structural unit of kappa- and iota-carrageenans is a disaccharide of β-D-galactose and 3,6-anhydro-α-D-galactose, while lambda-carrageenan does not contain 3,6-anhydrogalactose moieties [3,4]. The content of negatively charged sulfate ester groups varies, resulting in different gelling and thickening properties of carrageenans. Kappa-carrageenan has, on average, one sulfate group per disaccharide unit, while iota-carrageenan has two and lambda-carrageenan has three sulfate groups per disaccharide unit [5].

The use of carrageenans is due to their stabilizing, thickening, and gelling properties. Kappa- and iota-carrageenans interact strongly with proteins and are used as gelling agents in processed meat products [6,7] and as stabilizers in dairy products [8,9,10]. On the contrary, lambda-carrageenan has a weak interaction with milk proteins and is used mainly to increase viscosity and creaminess in milk desserts [4]. Carrageenan can also be found in other food products (e.g., in candies or jellies as a gelatin substitute), and in cosmetics (e.g., toothpastes as a thickener and stabilizer) and pharmaceuticals (e.g., as a thickening and moisturizing agent in throat preparations).

Industrial carrageenans vary widely in their dyad structure and degree of sulfation per repeating structural unit due to different macroalgal sources and extraction methods [11]. Therefore, the average number of sulfate groups often deviates from theoretical stoichiometry for a given carrageenan type, even in reagent-grade samples [12]. The presence of raw material residues and functional additives also reduces sulfate content. Typical additives include potassium chloride and locust bean gum, which are used to improve the gelling/thickening properties [13,14] as well as sucrose and glucose, which are used to standardize production batches [15].

The advanced instrumental techniques suitable for determining the degree of sulfation of carrageenan macromolecules include nuclear magnetic resonance (NMR) spectroscopy, Fourier-transform infrared (FTIR) spectroscopy, and high-performance liquid chromatography (HPLC). However, each of these methods has its limitations: ^1^H NMR spectroscopy is suitable for distinguishing the kappa, iota, and lambda subtypes [16]. The ^1^H and ^13^C NMR spectra, coupled in a two-dimensional technique, provide extensive information on the structure of repeating units and the positions of sulfate ester groups [17,18]. The technical limitations of NMR spectroscopy include low sensitivity for minor structural variants of the carrageenan backbone and overlapping signals from similar monosaccharide units. Moreover, restricted molecular mobility in solutions of high-molecular-weight carrageenans further worsens the resolution of spectral features. The presence of various counter-ions can shift resonance frequencies and distort peak shapes. High investment and maintenance costs of equipment and the staff experience required also limit the wide application of NMR spectroscopy in the carrageenan industry.

FTIR spectroscopy is useful to qualitatively indicate the presence of sulfate ester groups [19,20,21]. However, the key sulfate peaks are located in the wavenumber range from 1260 to 690 cm^−1^. This so-called fingerprint region contains many closely spaced characteristic bands from other structural units, which overlap with the sulfate ester bands and make it difficult to quantify the sulfate content. Multiple small differences in the structure of disaccharide units and the positions of sulfate groups cause the formation of shouldered peaks rather than distinct signals [22]. Residues of raw macroalgae matter and intentionally added carbohydrates and salts can further overlap or alter carrageenan vibrational bands. Moreover, moisture always present in carrageenans may disturb the shape of the critical vibrational bands. To improve the sulfate group quantitation, the absorbance of C–H bonds at 2920 cm^−1^ has been proposed as an internal standard [23]. However, a more straightforward approach requires using chemometric models [19,24].

HPLC is a versatile technique for analyzing the composition of carrageenan monomer units. For this purpose, it is necessary to carry out prior acid hydrolysis, which results in the formation of a mixture of individual monosaccharides. The usual hydrolysis methodology includes heating with concentrated trifluoroacetic acid (TFA) at 110 °C for 4 h [18,25,26,27]. The excess of TFA is neutralized by an alkali or removed by co-evaporation with methanol, and the hydrolysate obtained is derivatized with 1-phenyl-3-methyl-5-pyrazolone [18,25]. All these steps are labor-intensive and time-consuming, which limits the use of the HPLC technique in routine industrial analysis. Fortunately, the deep acid hydrolysis also breaks the sulfate ester bonds, and the resulting free sulfate anions can be quantified by ion chromatography or by nephelometry as barium sulfate [28]. A promising variant of chromatography for analyzing sulfated polysaccharides is ion-pair reversed-phase HPLC. The successful separation of carrageenan by HPLC-MS with a reversed-phase resin and hexylamine as the ion-pair reagent has been recently reported [29]. Carrageenan samples from the *Euchema* and *Gigartina* families were enzymatically degraded to oligomers before the analysis.

In addition to advanced instrumental techniques, there are also simple wet chemistry methods. These involve hydrolysis of a carrageenan sample followed by determination of the released sulfate anions. For turbidimetric determination, sulfate anions react with Ba ions in the presence of gelatin to stabilize the resulting suspension [28,30]. Ion chromatography can also be used for sulfate determination [31,32]. The hydrolysis step requires concentrated acid and prolonged heating (5–8 h).

Sulfate anionic charges can also be determined by ion-pair complexation with cationic dyes such as Methylene Blue, Toluidine Blue and Alcian Blue [12,30,33,34]. Among cationic dyes, Alcian Blue (AB) dye stands out due to its four cationic charges, which results in strong interaction with the anionic groups of carrageenan. The analytical method reported in [35] uses AB dye to precipitate carrageenan from aqueous solutions following by dissolution of the precipitate in monoethanolamine and photometry at 615 nm. The solubility of ion-pair complexes of AB dye with the anionic polymer poly(sodium styrene sulfonate) has been found to depend on the polymer-to-dye ratio [36]. Particle charge measurements have shown that ion-pair associate particles lose their colloidal stability at a nearly stoichiometric ratio of cationic to anionic charges [36]. 

In this paper, the concept of ion-associate precipitation at the stoichiometric charge ratio was used to determine sulfate groups in carrageenan. The working hypothesis is that Alcian Blue dye reacts with the sulfate groups of carrageenan in the same way as it reacts with the sulfate groups of poly(styrene sulfonate). A possible problem is that carrageenan macromolecules are much more hydrophilic compared to PSS and therefore the complexes formed may remain suspended in the reaction solution. Reference [35] reports that AB dye precipitates carrageenan from aqueous solutions in the form of an ion-pair complex. On the other hand, Reference [36] suggests the colloidal stability of ion-pair complexes of AB dye with PSS depends on the reactant ratio. To test the working hypothesis, several different types of carrageenans were tested in the reaction with AB dye. The objectives of the study were to select optimal experimental conditions, such as the concentrations and doses of carrageenan and AB dye and the time required for sedimentation. The advantage of the proposed procedure over the previously mentioned report [35] is that there is no need to use the toxic solvent monoethanolamine.

## 2. Results and Discussion

### 2.1. Formation of Ion Associates of Carrageenan with the AB Dye

#### 2.1.1. Qualitative Description of the Coagulation Phenomenon

**Figure 1:**
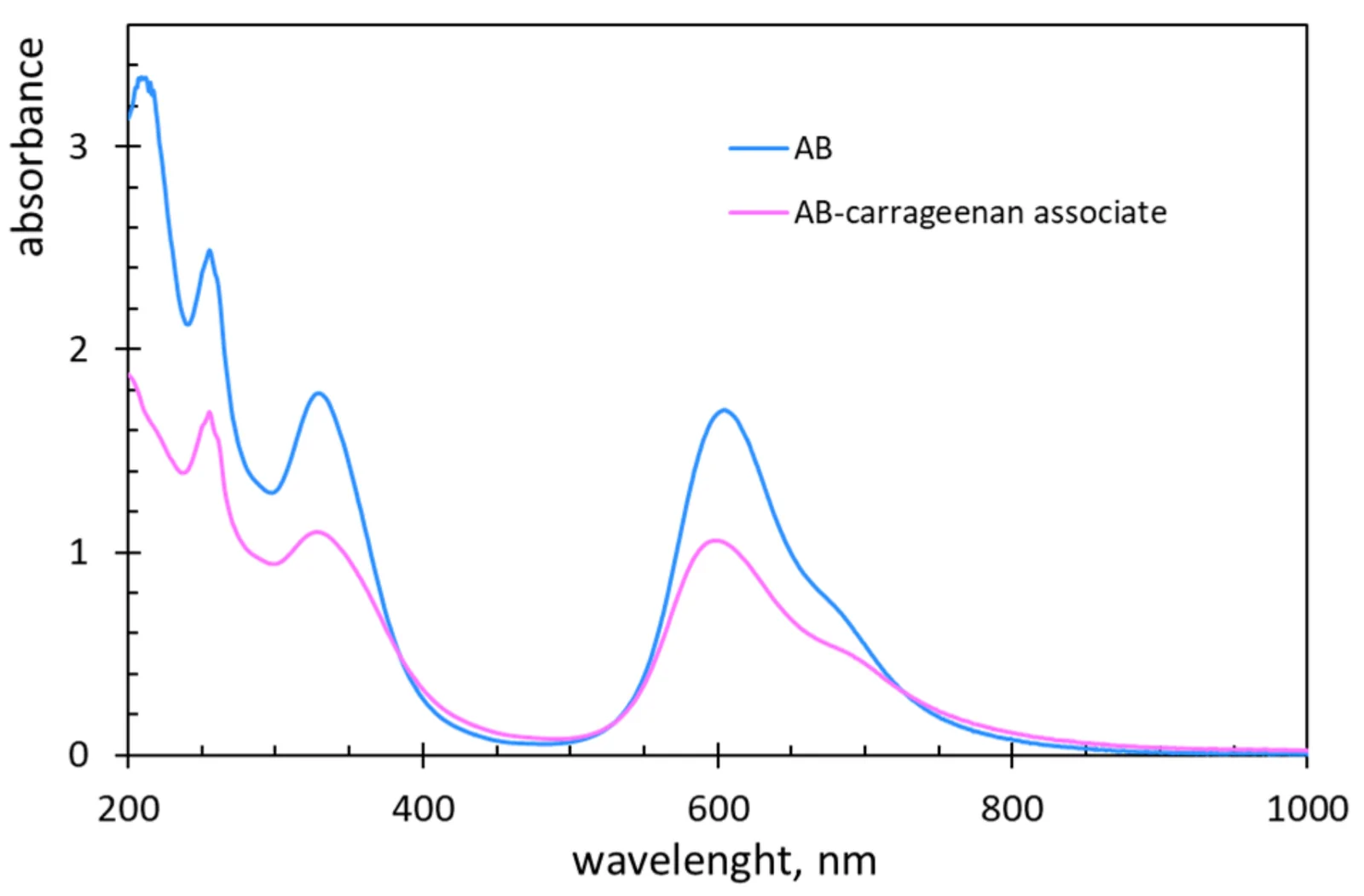
UV–vis spectra of the AB dye solution with the concentration of 95.9 mg/L (blue) and the colloidal solution of a mixture of κ-SA carrageenan with AB dye of the same concentration (pink). The spectrum of the carrageenan–AB mixture was recorded within 3 min after mixing.

Ion associates form immediately upon mixing carrageenan with the AB dye. Figure 1 shows the corresponding changes in the UV–vis spectra. The principal effect is that the main absorbance peaks of the dye decrease significantly. This is due to the dye transferring from the molecular solution to the colloidal solution. The peak absorbance in the vis region shifts by 7 nm to shorter wavelengths (Figure 1). This effect is called methachromasy and is often registered in cationic dyes associated with anionic polyelectrolytes [33,34,37,38]. On the contrary, absorbance values slightly increase in the ranges 400–470 nm and 750–850 nm, where the AB dye absorbance is low. This fact indicates the presence of microscopic insoluble particles of carrageenan–AB associates, which scatter light evenly at all wavelengths. The suspended particles tend to agglomerate, resulting in a change in their spectral characteristics. Therefore, the UV–vis spectra were recorded immediately after mixing.

**Figure 2:**
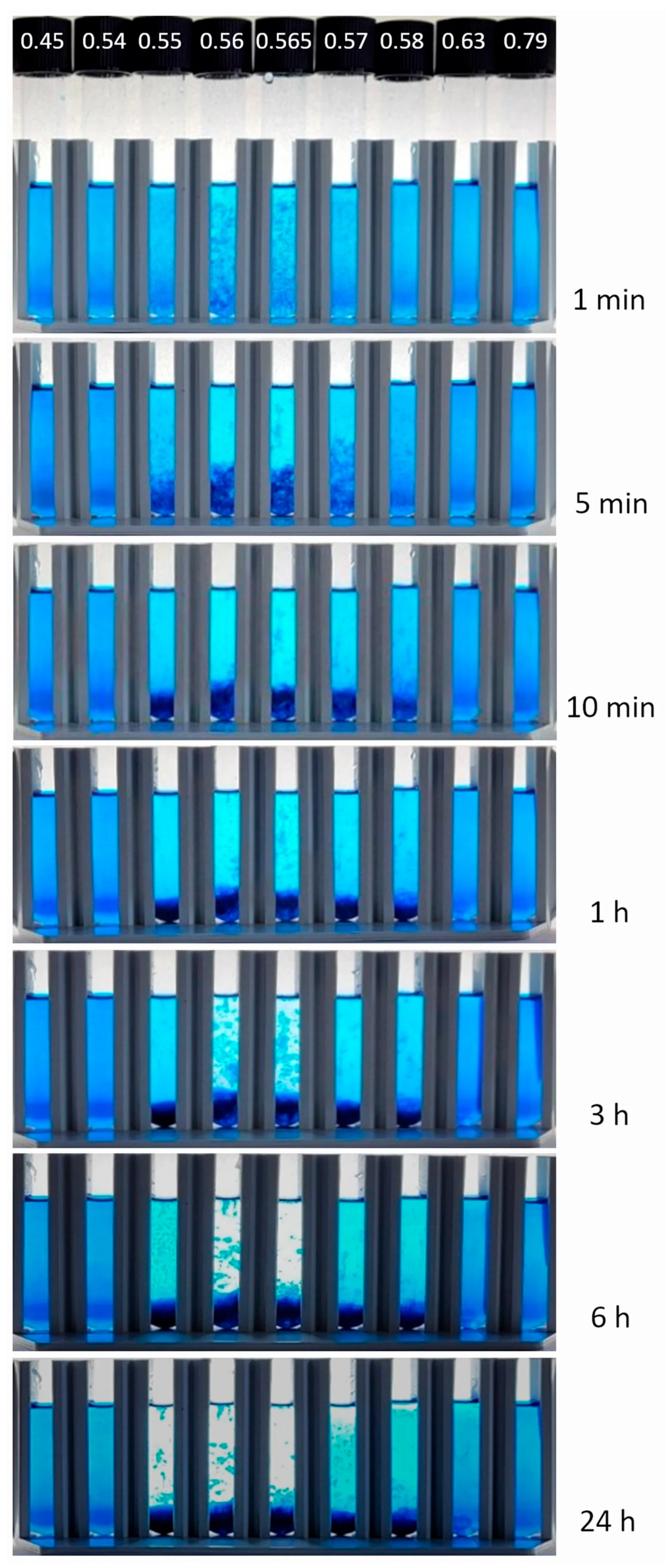
Series of mixtures of carrageenan κ-SA with AB dye at a concentration of 383.4 mg/L, with the indicated values of the mass ratio of the components (g/g).

The particulate ion associates formed tend to bind together and form larger aggregates, which lose colloidal stability and sediment slowly. Carrageenan is known to coagulate with organic cations, such as hexadecyltrimethylammonium [39], choline [40], and poly(diallyldimethylammonium chloride) [41]. Figure 2 shows the images of mixtures of κ-SA carrageenan with AB dye taken at consecutive times. The photos clearly show that binding carrageenan with the AB dye results in intense coagulation at a specific carrageenan-to-dye ratio. The range of component mass ratios to form insoluble associates appears to be quite narrow and is 0.56–0.565 g/g. Outside this range, coagulation is rather partial (at 0.55 g/g and 0.57–0.58 g/g), and a part of the AB dye remains in the solution (Figure 2). When the carrageenan-to-dye ratios are equal to or lower than 0.54 g/g and equal to or higher than 0.63 g/g, the resulting suspensions are fully stable and do not contain sediments. Similar behavior was observed in the mixtures of the AB dye with poly(styrene sulfonate) [36].

The sample with a carrageenan-to-dye ratio of 0.565 g/g is the most unstable. It begins to precipitate immediately upon mixing the components (Figure 2). The precipitate removes most of the dye molecules from the solution, resulting in an almost colorless supernatant. The complete binding of the dye by carrageenan indicates an equivalent number of active cationic groups of the dye and anionic groups of carrageenan. The deviation of the carrageenan-to-dye ratio from the critical value of 0.565 g/g results in a reduction in the amount of precipitated solid and an increase in the color of the supernatant (Figure 2).

The mechanism of ion-associate coagulation has been recently described for the association of carrageenan with cetyltrimethylammonium [42] and of PSS with AB dye [36]. Zeta potential measurements revealed that loss of solubility and rapid coagulation occur when the amounts of opposite charges on the cationic dye and anionic polymer are nearly equal [36]. Due to charge neutralization, the ion-associate particles become hydrophobic and form large aggregates. Therefore, the sizes of ion-associate particles are maximal just at the equimolar ratio of cationic and anionic charges [36].

**Figure 3:**
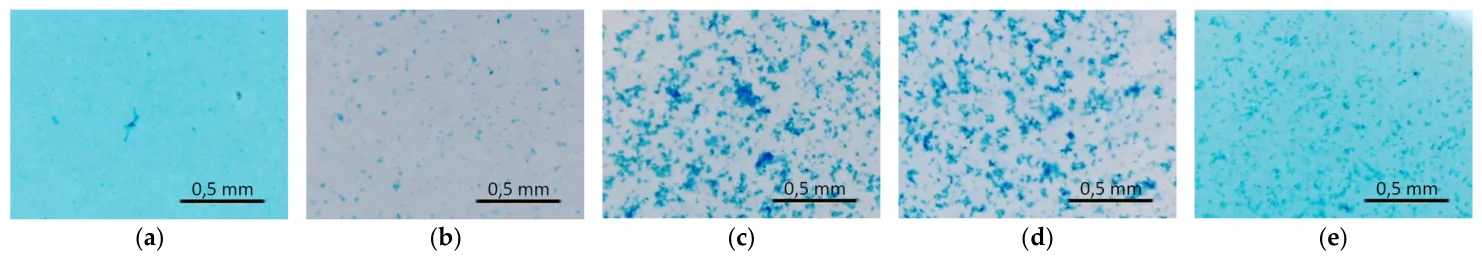
Microscopic images of selected samples of a series of mixtures of κ-SA carrageenan with AB dye at a concentration of 383.4 mg/L, with the following values of the mass ratio of the ingredients (g/g): (**a**) 0.45, (**b**) 0.55, (**c**) 0.565, (**d**) 0.58 and (**e**) 0.79.

The microscopic images in Figure 3 give additional insight into flocculation of the carrageenan–AB associates. The image of a suspension with a low carrageenan-to-dye ratio of 0.45 revealed a few small particles ranging in size from 0.03 to 0.08 mm and single agglomerates 0.2 mm long (Figure 3a). The blue background is from excess dye remaining in the soluble state. In the sample with a carrageenan-to-dye ratio of 0.55 g/g, the number of particles is significantly higher (Figure 3b), but their sizes remain small, i.e., from 0.03 to 0.08 mm. The excess positive charges in the particles prevent them from associating into larger flocs, so the precipitation is rather partial (Figure 2). In the sample with a carrageenan-to-dye mass ratio of 0.565 g/g, the precipitate particle size is by far the largest in the entire series and reaches 0.33 mm (Figure 3c). Due to intense agglomeration, it is difficult to even distinguish the molecules of the associate. Accordingly, the coagulation at this carrageenan-to-dye ratio is the most intense (Figure 2). Further increasing the proportion of carrageenan up to a value of 0.79 g/g causes the number and the size of suspended particles to decrease significantly (Figure 3d,e). The obvious cause is that an excess of carrageenan macromolecules stabilizes colloidal carrageenan-dye associates. Therefore, at the highest carrageenan-to-dye mass ratio, the background is again blue (Figure 3e) and the ability of the associate to sediment disappears (Figure 2).

#### 2.1.2. Determination of the Critical Carrageenan-to-Dye Ratio

**Figure 4:**
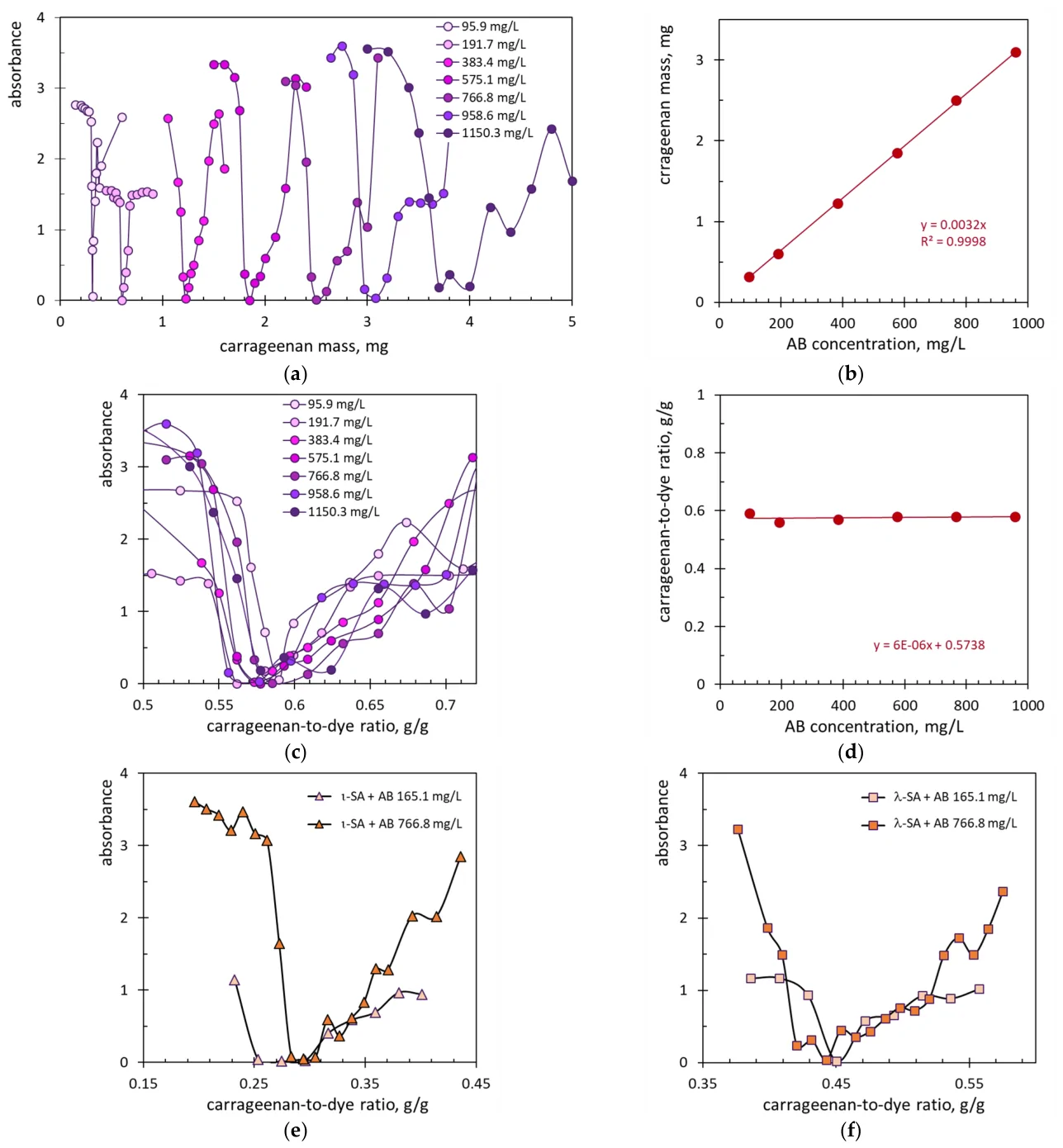
(**a**) The absorbance of supernatant at 600 nm vs. the κ-SA carrageenan mass added to 5 mL of AB solutions with the indicated concentrations. (**b**) The mass of κ-SA carrageenan causing the maximum sedimentation vs. the concentration of the AB dye. (**c**). Absorbance of supernatant vs. the carrageenan-to-dye mass ratio determined for the κ-SA sample. (**d**) The critical carrageenan-to-dye mass ratio vs. the concentration of the AB dye determined for the κ-SA sample. (**e**) Absorbance of supernatant vs. the carrageenan-to-dye mass ratio determined for the ι-SA samples under conditions analogous to those for κ-SA. (**f**) Absorbance of supernatant vs. the carrageenan-to-dye mass ratio determined for the λ-SA samples under conditions analogous to those for κ-SA.

The quantitative insight into the association and coagulation of carrageenan with AB dye was obtained by spectrophotometric measurements. Mixtures with varying dye concentrations and carrageenan-to-dye mass ratios were prepared and left for sedimentation for 6 h. Figure 4a shows the absorbance values of the supernatants. The registered fluctuations in absorbance are due to ion-associate flocs remaining partially suspended. Despite the absorbance fluctuations, a clear minimum is registered in each series, which corresponds to the deep sedimentation of the formed ion associate (Figure 4a). The only exception is the plot obtained at the dye concentration of 1150.3 mg/L: the minimum absorbance values were recorded at carrageenan masses of 3.64 and 4.02 mg. Therefore, it is not recommended to use working dye solutions with a concentration above 1000 mg/L.

Figure 4b shows the mass of carrageenan in the sample corresponding to the minimum absorbance versus the mass of AB dye present in the sample. The straight plot indicates clearly that the product of ion association of carrageenan with the AB dye has a constant composition.

Figure 4c presents the same data on supernatant absorbance as in Figure 4a, but plotted against the ratio of the components. All the plots have minima at the same carrageenan-to-dye ratio. Thus, Figure 4d confirms that the critical carrageenan-to-dye ratio remains stable at different concentrations of the AB dye.

Figure 4e shows the values of supernatant absorbance plotted against the carrageenan-to-dye mass ratio for iota-carrageenan at different concentrations of AB dye. Again, clear minima are registered at the same carrageenan-to-dye mass ratios independent of the concentration of dye solution. Figure 4f provides the same inference for lambda-carrageenan.

#### 2.1.3. FTIR Spectra of Ion Associates of Carrageenan with the AB Dye

**Figure 5:**
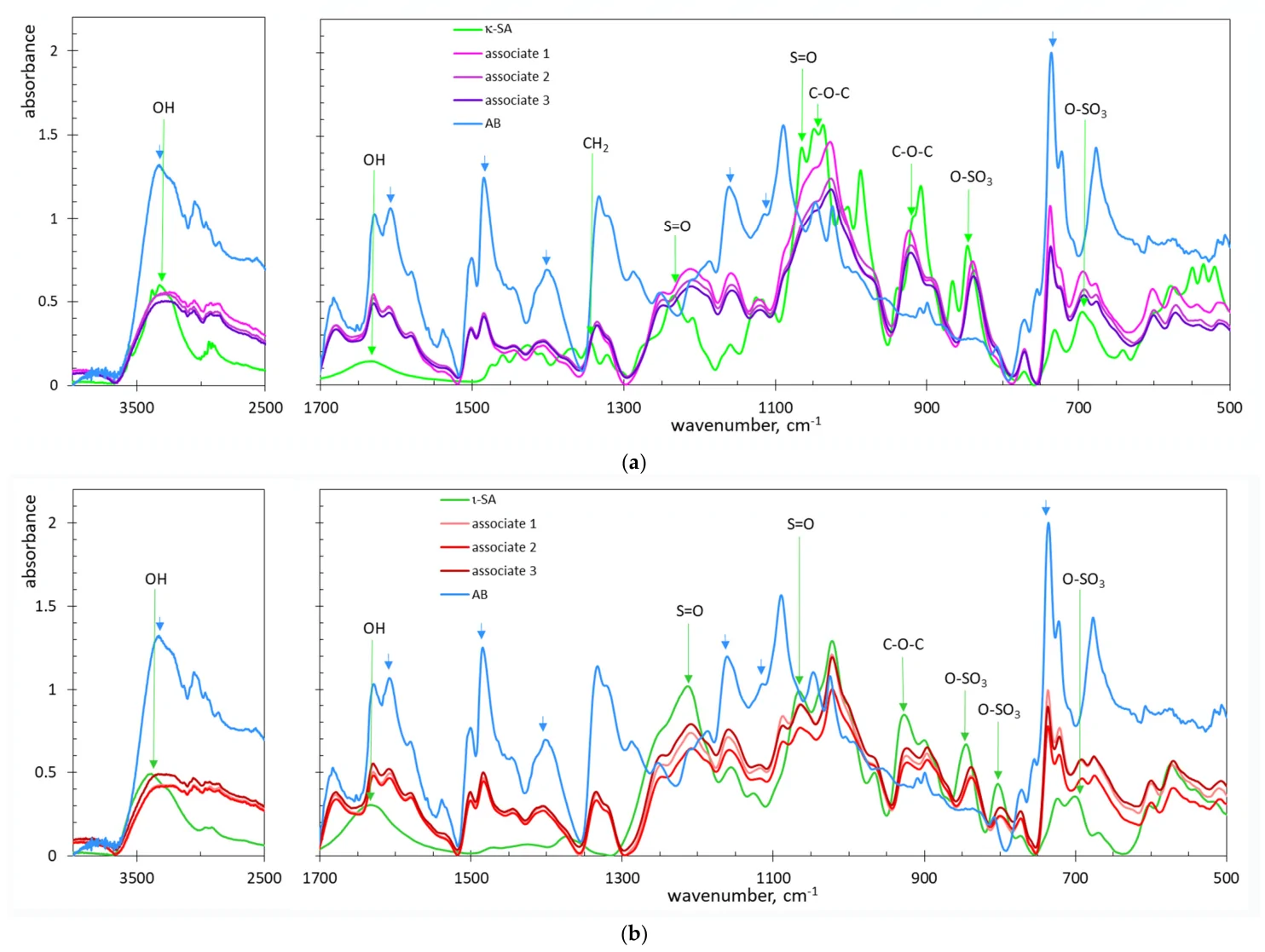

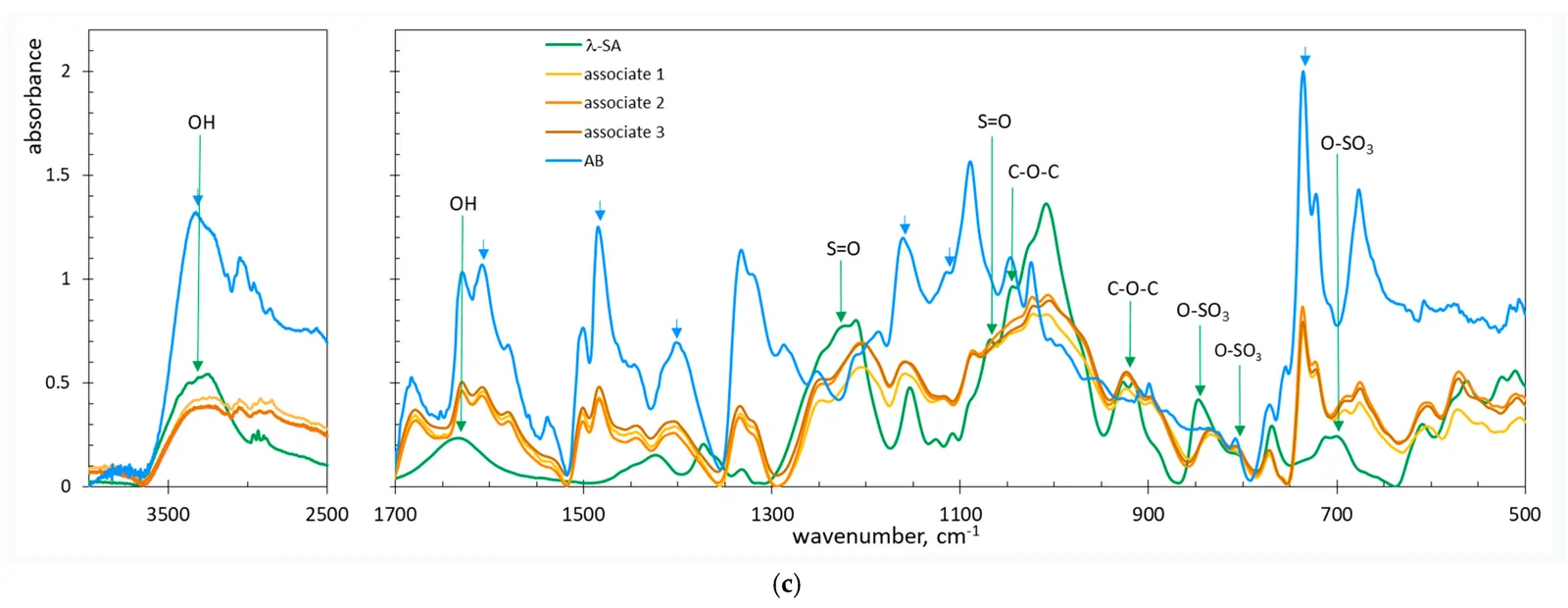
FTIR spectra of ion associates of κ-SA (**a**), ι-SA (**b**), and λ-SA (**c**) carrageenans with the AB dye precipitated from solutions of dye concentrations: 383.4 mg/L (associate 1), 766.8 mg/L (associate 2), and 1150.3 mg/L (associate 3). FTIR spectra of the starting carrageenans and AB dye are given for comparison. Arrows indicate characteristic bands of the dye (blue) and carrageenans (green).

Figure 5 shows ATR-FTIR spectra of the dried precipitates along with the spectra of carrageenans of different types and AB dye. The main bands are summarized in Table 1. The FTIR spectrum of Alcian Blue dye contains a broad band at 3400–3100 cm^−1^ and sharp peaks at 1610, 1486, 1390, 1151, 1102, and 735, which can be attributed to N-substituted pyridinium. The FTIR spectra of carrageenans contain absorbance bands characteristic of sulfated polysaccharides: 3400 and 1640 cm^−1^ (OH groups); 1220, 1060, 840, and 697 cm^−1^ (ester sulfate groups); and 920 and 1046 cm^−1^ (C–O–C bonds). The spectra of iota-carrageenan samples also contain a moderate peak at 800 cm^−1^, which is attributed to –O–SO_3_ stretching vibrations. The presence of this signal in the spectrum of carrageenan λ-SA suggests the presence of an admixture of iota-type carrageenan. The FTIR spectra of the carrageenan-dye associates contain the bands characteristic of both carrageenan and the AB dye (Figure 5). No additional peaks are registered, proving that the precipitate is an ion associate rather than a new chemical compound. The spectra of the ion associates precipitated from solutions with different dye concentrations are nearly identical (Figure 5a–c). This indicates that all the precipitates have very similar chemical composition.

### 2.2. Application of Ion-Associate Sedimentation for Analytical Purposes

The formation of carrageenan–AB associates can be used to determine the content of sulfate groups in the carrageenan samples and/or the quality of commercial carrageenans. This method includes a serial titration of the AB solution with the carrageenan solution and recording the carrageenan-to-dye ratio that yields maximal coagulation. The intense blue color of the AB dye is favorable for visual registration of the critical point (compare Figure 2). In turn, the presence of four positive charges ensures strong binding of Alcian Blue to carrageenan. However, it should be taken into account that the number of active cationic charges in the AB dye differs from the theoretical one [36]. The obvious reason is the presence of fillers usually added to commercial textile dyes. Therefore, an AB solution for analytical application should be thoroughly standardized. Another technical aspect to address is the time required for reliable detection of sedimentation. The optimal time depends on the concentration of the AB dye that was used.

#### 2.2.1. Standardization of the Alcian Blue Solution

**Figure 6:**
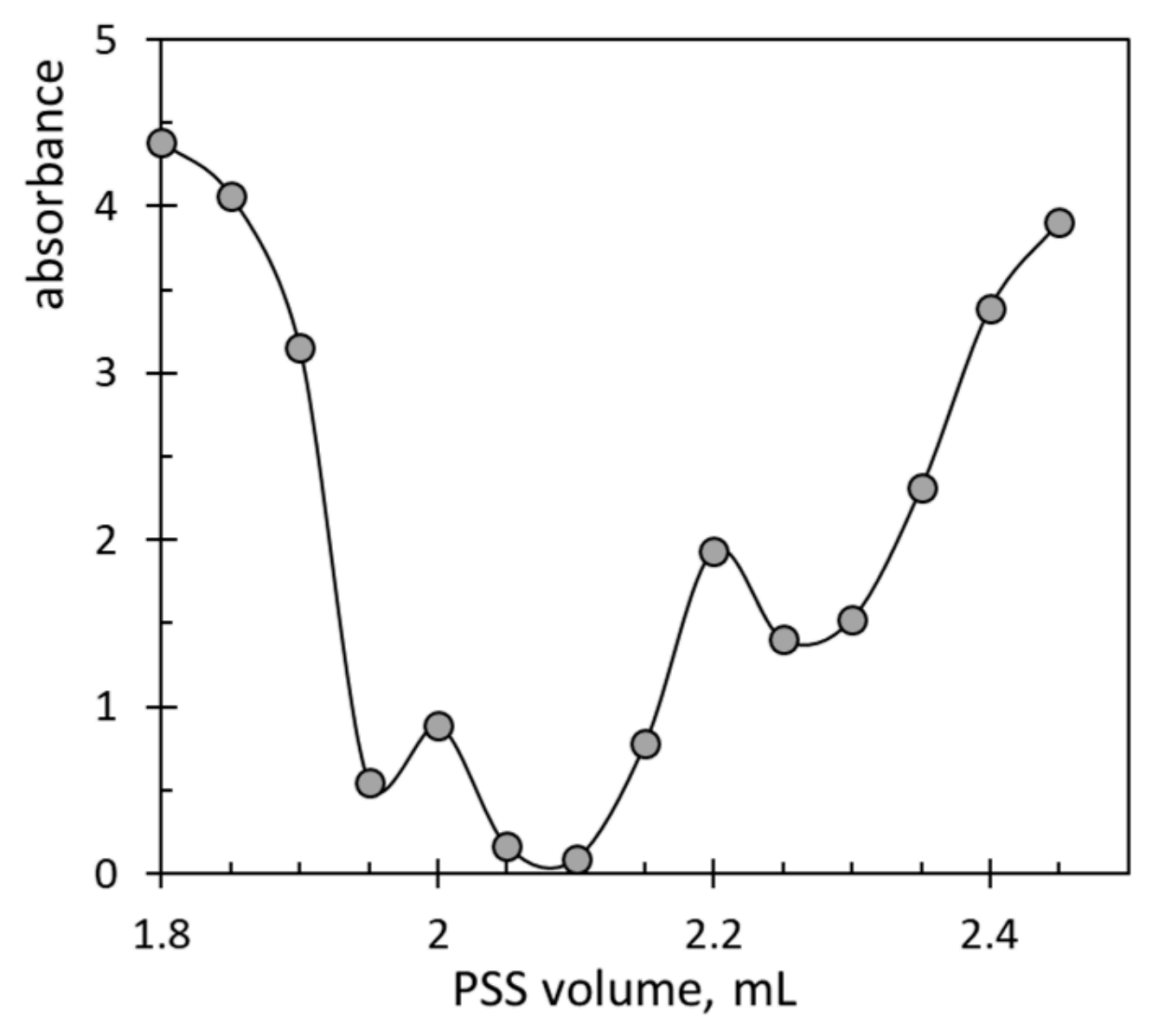
Absorbance of the supernatant was measured for 5 mL of the AB dye solution mixed with a stock solution of PSS at different volumes of added PSS solution. The concentrations of dye and PSS are 800 mg/L and 2.5 mM, respectively.

To standardize AB solutions, the formation of an ion associate with poly(styrene sulfonate) (PSS) was used. Unlike naturally derived carrageenan, PSS is a synthetic polymer with a constant and repeatable composition, which enables its use for AB standardization. The deep sedimentation occurs in a narrow window of the dye–PSS ratio, which allows for visual identification of the equilibrium point [36]. Therefore, coagulation of AB dye with standard PSS solution was used to determine the actual concentration of AB dye. Figure 6 shows the results of absorbance measurements for a series of PSS samples mixed with an 800 mg/L AB solution. The oscillations in the curve originate from sediment particles that remain suspended in the solution.

Figure 6 clearly indicates that a minimum absorbance is observed when 2.1 mL of 2.5 mM PSS is mixed with 5 mL of AB dye at a concentration of 800 mg/L. These values enable the calculation of the content of active cationic charges in the commercial dye sample, which was found to be 1.3 mmol/g.

#### 2.2.2. Optimal Time of Sedimentation

**Figure 7:**
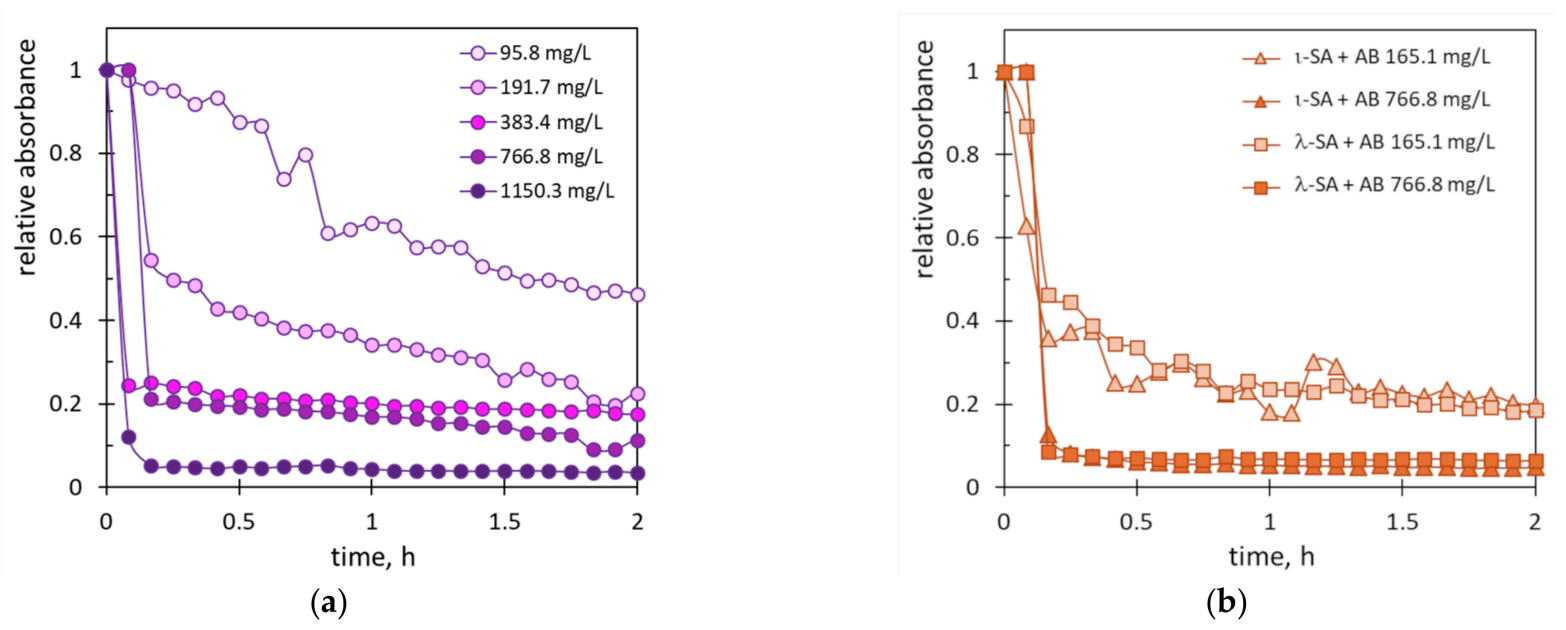
Kinetic lines of sedimentation in the mixtures of κ-SA (**a**) and ι-SA and λ-SA (**b**) carrageenans with AB dye. The mass ratio of components is 0.565, 0.29, and 0.44 g/g for κ-SA, ι-SA, and λ-SA samples, respectively. The dye concentrations are indicated in the legend.

Figure 7 shows the relative change in absorbance recorded at 600 nm in mixtures containing various doses of the dye and carrageenan κ-SA at a mass ratio of 0.565 g/g. For low reagent concentrations, sedimentation lasted from 5 to 20 h. With increasing dye and carrageenan concentrations, the sedimentation time shortened to 20 min in the tested concentration range. This is due to the higher load of components resulting in a larger number of particles, which favors intense flocculation.

A similar effect was observed for the coagulation of PSS with AB dye. At the optimal PSS-to-AB ratio and a dye concentration of approximately 834 mg/L, sedimentation took approximately 3 min [36]. The faster sedimentation of PSS-AB associates is due to their more hydrophobic character. On the contrary, carrageenan macromolecules contain multiple OH groups, which promote a solvation shell and thereby prevent flocculation of their ion associates.

#### 2.2.3. Reproducibility and Linear Range of Sulfate Group Determination

The reproducibility experiments were carried out using the κ-SA carrageenan and the AB dye solution of 165.1 mg/L. For a series of seven repetitions, the following parameters were determined: the average value of the critical carrageenan-to-dye ratio was 0.56 g/g, variance was 0.00035, standard deviation was 0.0187 g/g, and relative standard deviation was 3.34%.

In the range of carrageenan dose from 0.28 to 3.3 mg per 5 mL of the dye solution, the relationship between carrageenan mass and the critical AB mass is linear with a correlation coefficient of 0.9981.

#### 2.2.4. Examples of Determination of Sulfate Groups in Carrageenans

**Figure 8:**
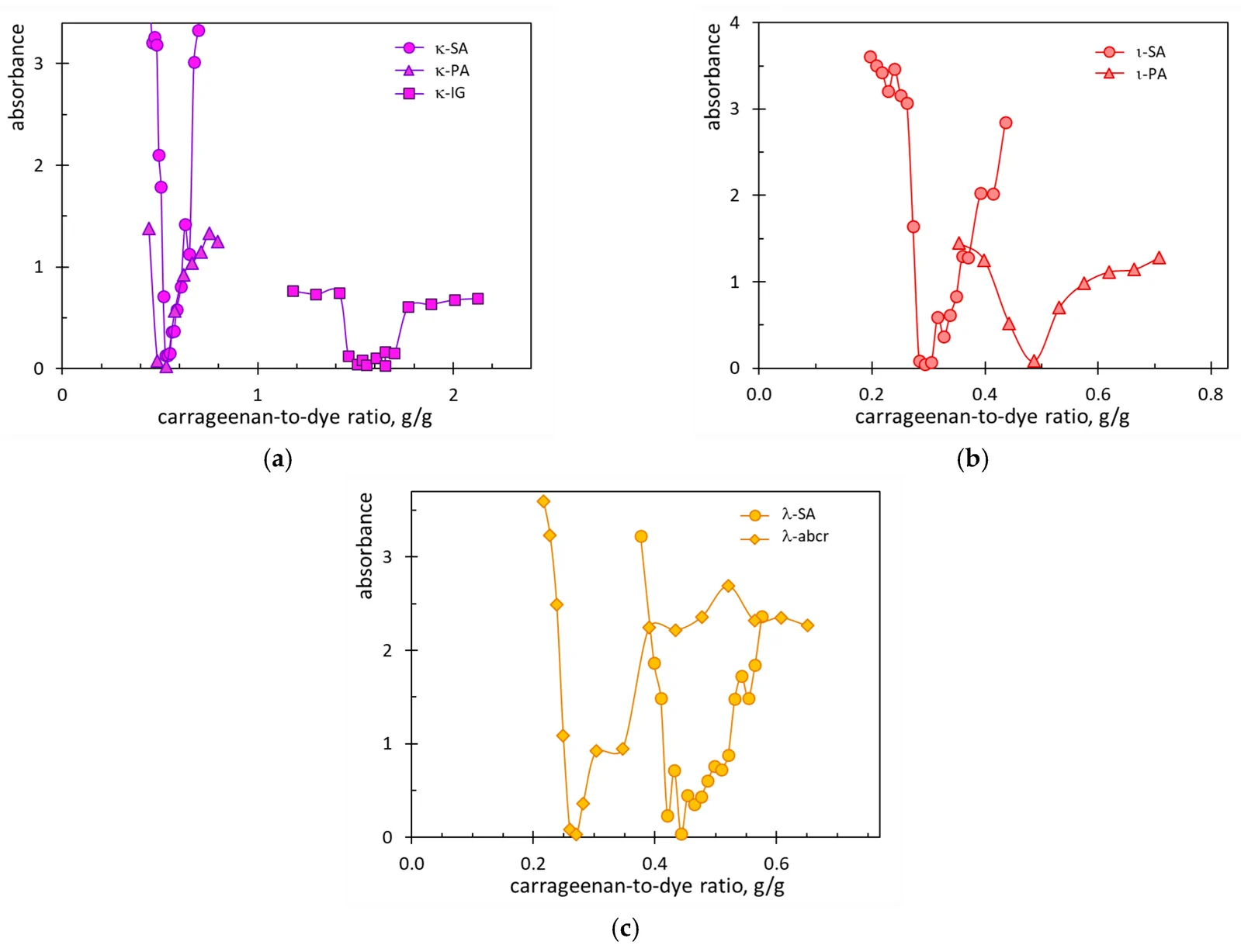
Absorbance of the supernatant in the mixtures of carrageenan and AB dye solutions depending on the carrageenan-to-dye mass ratio. The carrageenan samples (indicated) were of (**a**) kappa, (**b**) iota, and (**c**) lambda type. The concentration of AB dye solution was 800 mg/L.

A series of commercial carrageenans of the kappa, iota, and lambda types were tested. The samples came from different manufacturers, and the carrageenan content of a given type in the product was generally not precisely determined. For each product, serial titrations of the AB dye were carried out. After sedimentation for 6 h, the absorbance of the supernatant was measured to determine the equimolar charge ratio of carrageenan and the dye. Figure 8 shows the measurement results.

The shapes of the curves in the figures indicate significant differences in the composition of the carrageenans tested. In the case of κ-SA and ι-SA carrageenans (Figure 8a,b) and λ-abcr carrageenan (Figure 8c), the minimum was recorded at lower carrageenan-to-AB mass ratios compared to carrageenans from other manufacturers. This indicates a higher sulfate content compared to other carrageenan samples. On the contrary, the masses of the κ-IG and ι-PA carrageenan samples required to neutralize the dye charges are the highest (Figure 8a,b). This indicates a decreased content of ester sulfate groups.

**Figure 9:**
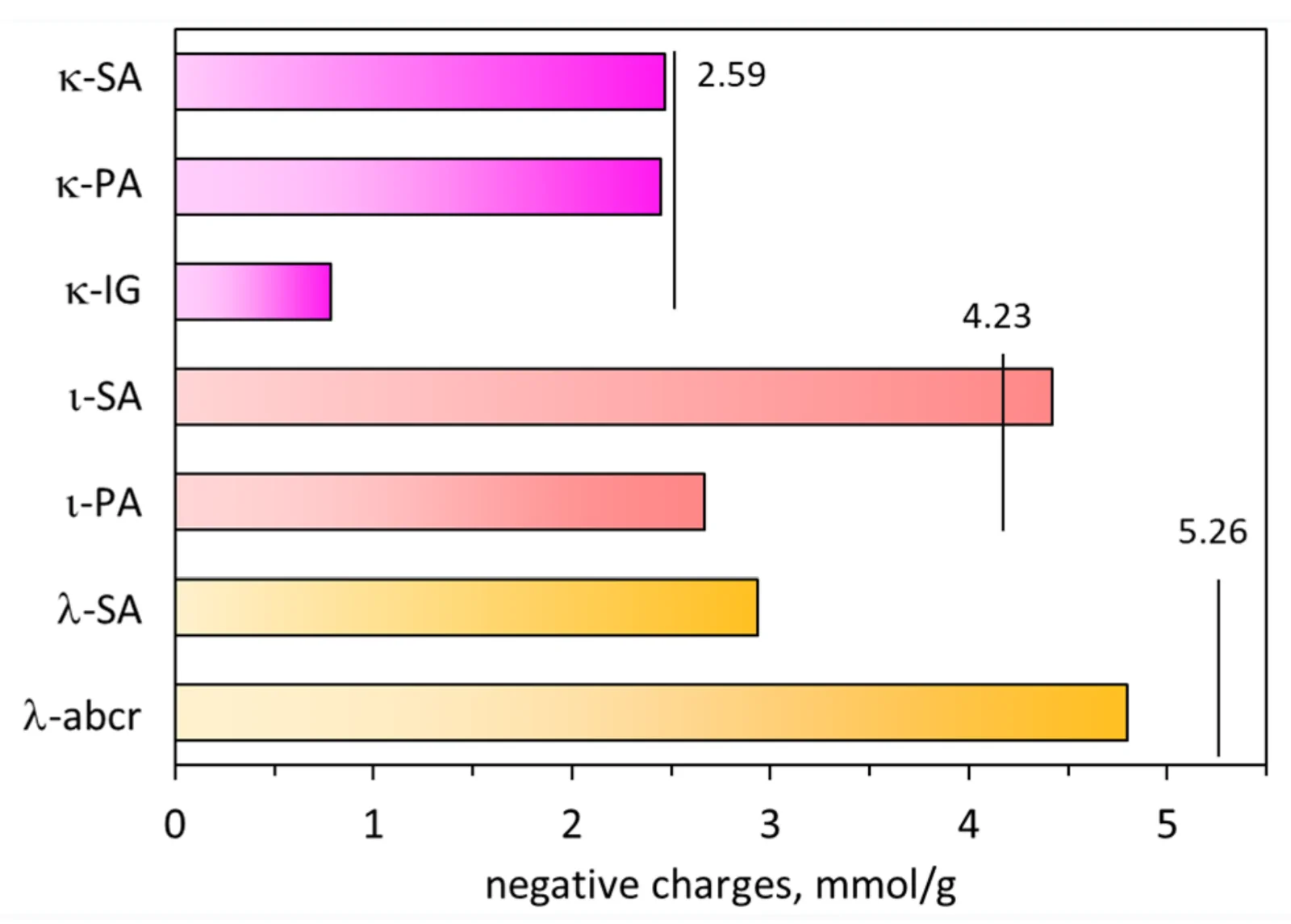
The content of anionic groups in the tested carrageenan samples. The lines indicate the theoretical content of sulfate groups in kappa, iota, and lambda carrageenans, which is 2.59, 4.23, and 5.26 mmol/g, respectively.

Based on the equimolar charge ratio between the dye and carrageenan, the number of negative charges of carrageenan required to neutralize the dye charges was determined. Figure 9 shows the results obtained as the contents of sulfate groups in mmol per gram of carrageenan samples.

The idealized number of sulfate groups per structural disaccharide unit is 1 for kappa carrageenan, 2 for iota carrageenan, and 3 for lambda carrageenan. Correspondingly, the theoretical content of sulfate groups is 2.59, 4.23, and 5.26 mmol/g for kappa-, iota-, and lambda-carrageenans, respectively. Figure 9 shows that the measured content of sulfate groups in the tested carrageenan samples differ from the theoretical ones. The obvious explanation is that commercial samples rarely possess an ideal structure. A lower content of sulfate groups compared to the theoretical value may also be caused by the presence of impurities or fillers.

Among kappa-carrageenans, the κ-SA and κ-PA samples are characterized by the highest content of sulfate ester groups, close to the theoretical value (Figure 9). In contrast, the κ-IG sample contains a much smaller content of sulfate groups (Figure 9), suggesting the presence of fillers. This explanation is supported by the fact that the solutions of the κ-IG carrageenan sample are slightly turbid, indicating the presence of insoluble particles.

The iota-carrageenans also differ in their sulfate content (Figure 9). It was found that the content of sulfate groups in the ι-SA carrageenan is higher than the theoretical value. The only reason is the discrepancy between the molecular structure of this sample and the ideal structure of iota-carrageenan. In contrast, the ι-PA sample has a lower content of sulfate groups (Figure 9). Its solutions are visibly turbid, suggesting the presence of impurities or admixtures.

Both lambda carrageenan samples showed lower sulfate group content compared to the theoretical value (Figure 9). In the case of lambda carrageenans, low-molecular-weight carbohydrate diluents are often added to standardize the viscosity of production batches [15].

The values of sulfate content obtained (Figure 9) turned out to be markedly higher than the sulfate content determined by the classical method using acid hydrolysis followed by turbidimetry (Table 3). The calculated recovery values range from 105 to 119%.

#### 2.2.5. FTIR Spectra of the Tested Samples

**Figure 10:**
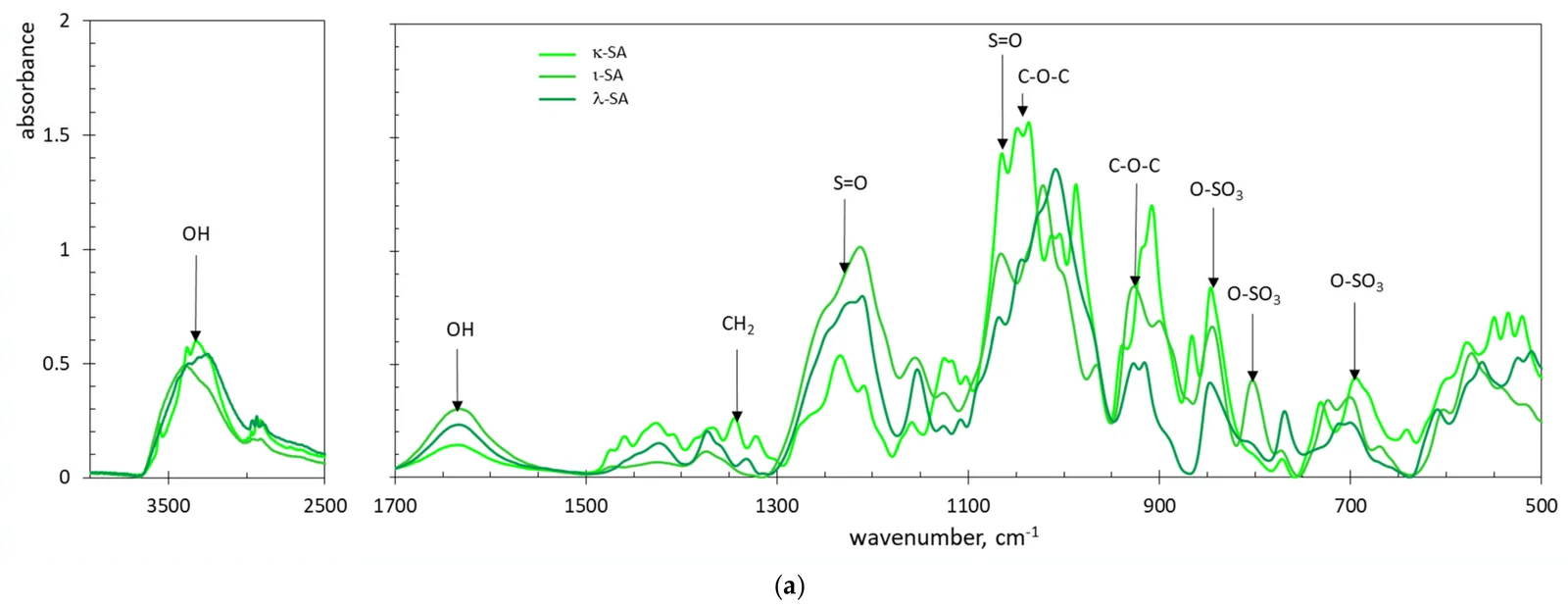

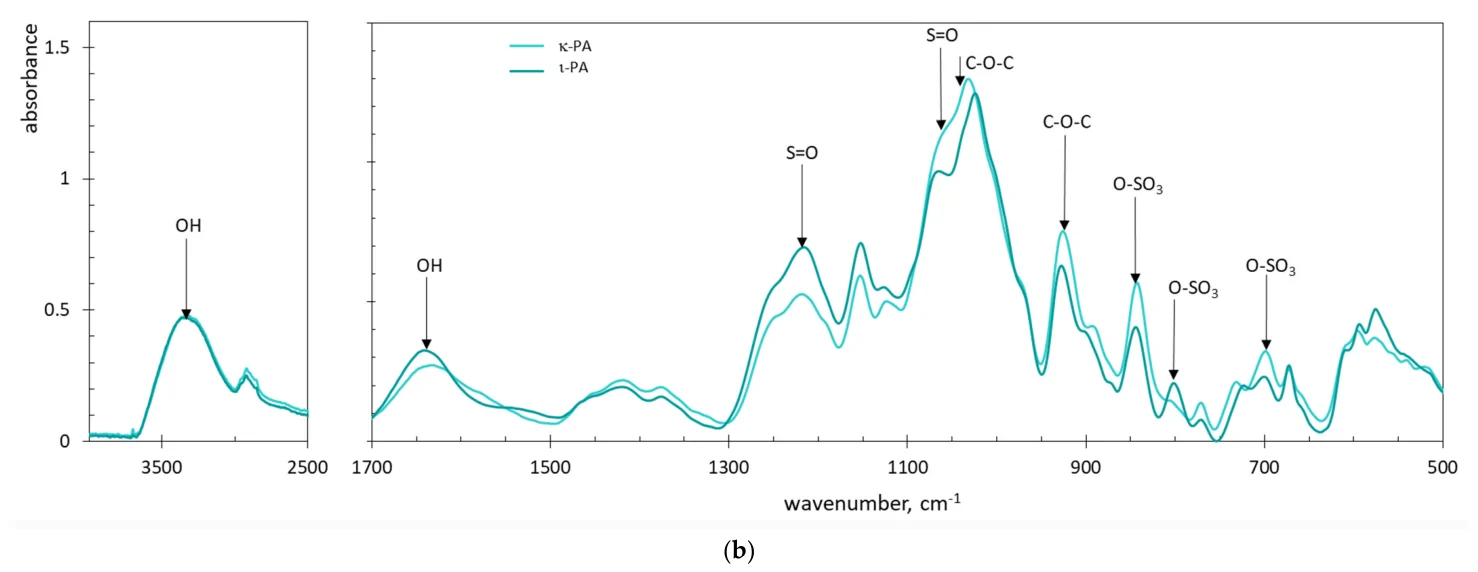
FTIR spectra of carrageenans of the SA (**a**) and PA (**b**) series.

To explain the dye titration results, the FTIR spectra of the tested carrageenan samples were analyzed (Figure 10).

Of the four bands associated with the presence of sulfate groups (negative charge carriers), the number of these groups is best represented by the band intensity at approximately 1210 cm^−1^. Figure 10a shows FTIR spectra of carrageenans in the SA series. The band at 1210 cm^−1^ in the spectrum of the ι-SA sample is approximately twice as intense as that of the κ-SA sample. This is consistent with the theoretical structure of iota- and kappa-carrageenans. In turn, the band at 1210 cm^−1^ in the spectrum of the lambda-carrageenan sample from the SA series is significantly lower than that expected (Figure 10a). This product likely contains a significant amount of other types of carrageenan. Usually, extraction of pure lambda-carrageenan requires selecting a proper algae genus and plant-life stage. Another common cause may be impurities of non-sulfated polysaccharides present due to incomplete purification.

In the PA series of carrageenans, the difference in the absorbance at 1210 cm^−1^ is smaller than that expected from the theoretical structures of kappa- and iota-carrageenans (Figure 10b). In this case, iota-carrageenan likely contains an admixture of kappa-carrageenan or other polysaccharides.

**Figure 11:**
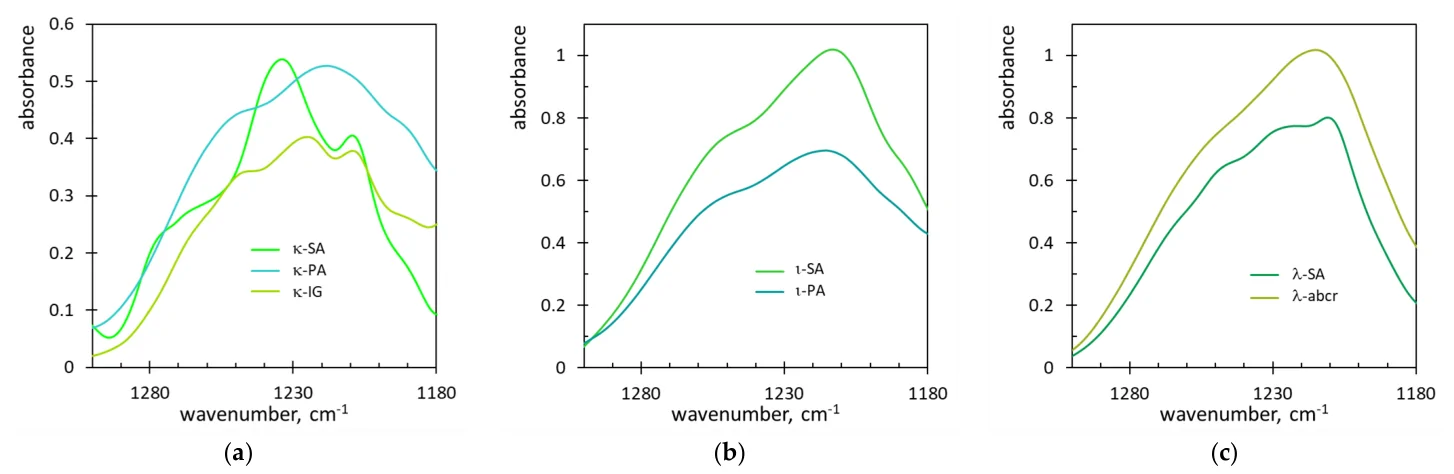
Bands at approximately 1210 cm^−1^ in the FTIR spectra of carrageenans from different producers, depending on the type: (**a**) kappa-carrageenans, (**b**) iota-carrageenans, (**c**) lambda-carrageenans.

Figure 11 shows the comparison of the band at 1210 cm^−1^ in the FTIR spectra of carrageenans of a given type from different suppliers. Among the kappa carrageenans, the κ-SA and κ-PA samples are characterized by similar anionic group content (Figure 11a). This is consistent with the determined content of anionic groups (Figure 9). The κ-IG carrageenan has the lowest band at 1230–1210 cm^−1^ and the lowest content of anionic groups among kappa-carrageenans (Figure 9 and Figure 11a). In the case of iota-carrageenans, the difference between the ι-SA and ι-PA samples is similar in terms of band intensity at 1230–1210 cm^−1^ and in the content of anionic groups (Figure 9 and Figure 11b). In the group of lambda-carrageenans, the λ-abcr sample is characterized by higher band intensity at 1230–1210 cm^−1^ and higher content of anionic groups (Figure 9 and Figure 11c).

**Figure 12:**
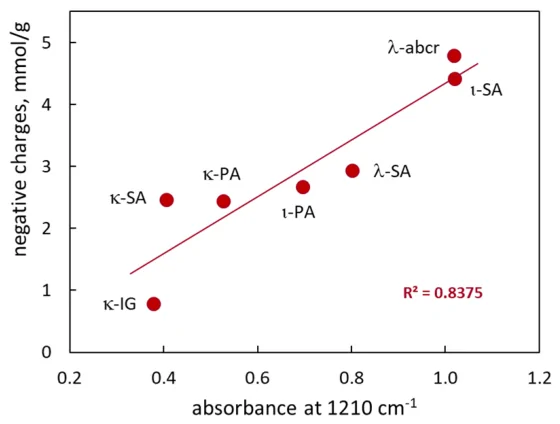
The relationship between the content of sulfate groups in carrageenan determined with the aid of AB dye and the band height at 1210 cm^−1^ in the FTIR spectra of the tested carrageenan samples (Figure 11).

Figure 12 shows the relationship between the determined content of sulfate groups (mmol/g) and the absorbance at 1210 cm^−1^, which is characteristic of sulfate groups. It indicates that the number of sulfate groups determined with the AB dye correlates with the absorbance at 1210 cm^−1^. The correlation is not entirely straightforward (R^2^ = 0.8375), but in the case of ATR-FTIR spectral data, it is quite satisfactory (Figure 12). This confirms that the determination of sulfate groups by association with the AB dye is a reliable analytical method.

#### 2.2.6. Compliance with the Green Analytical Chemistry Concept

**Figure 13:**
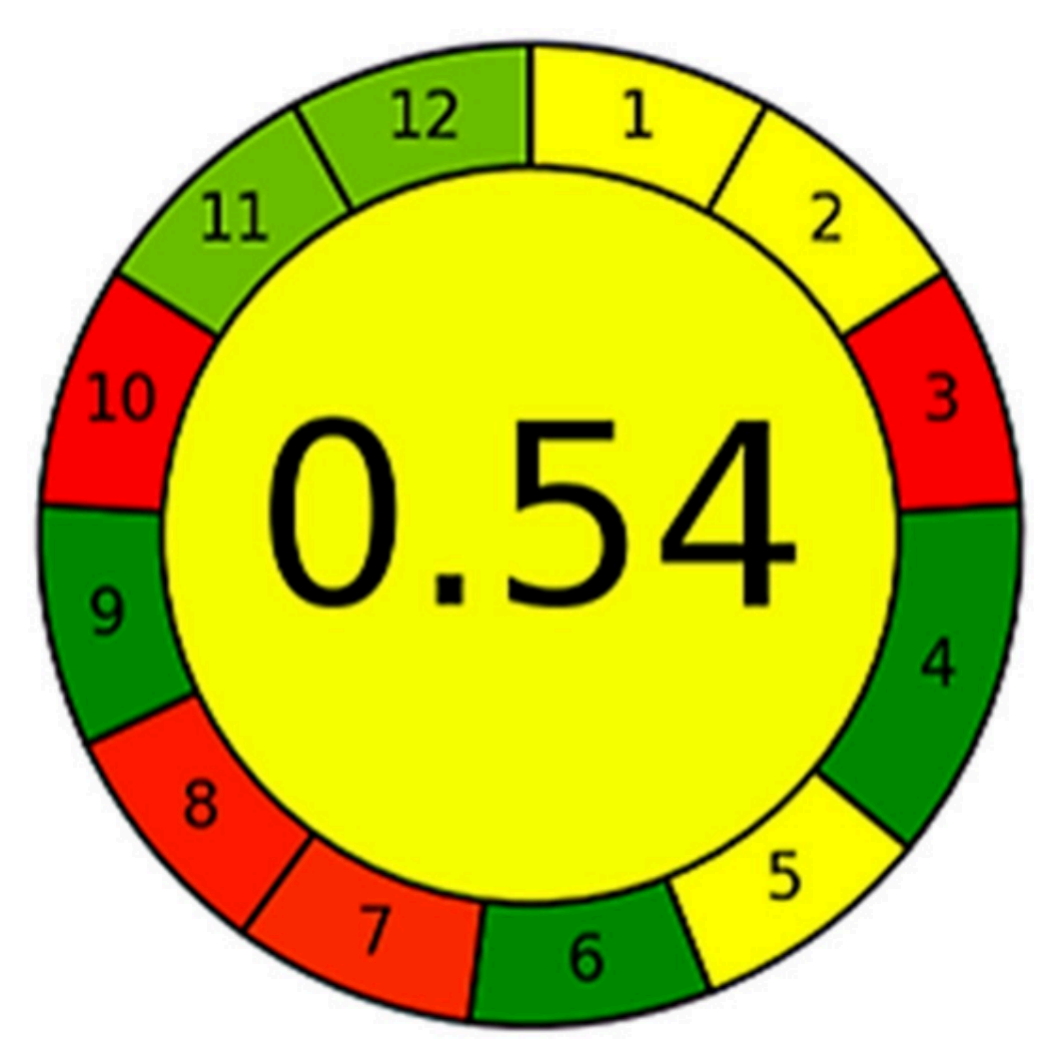
AGREE test result for the method of determining sulfate groups in carrageenans using Alcian Blue dye. The greenness of individual criteria and resultant rating is marked with smoothly changing colors (from green—the best result to red—the worst result).

In fact, the proposed method is a titration procedure. As with other titration methods, no compressed gases or toxic solvents are used, and electricity consumption is very low. The greenness of the method was evaluated using the AGREE test [48]. Table 2 lists the evaluation criteria, and Figure 13 shows the resulting diagram.

The AGREE test indicates that the method’s greatest advantage is its simplicity. The sample requires no preparation, and the procedure—requiring only a single reagent—can be performed without the use of instrumentation. This results in maximum scores for parameters 4, 6, and 9, marked in green in Figure 13. An additional benefit is the low level of operator exposure, reflected in the high values of parameters 11 and 12. On the other hand, the proposed method is characterized by low efficiency and generates a relatively large volume of waste, as indicated by the low values of parameters 3, 8, and 7, shown in red in the diagram (Figure 13). From the point of view of the greenness of the method, the use of a reagent not coming from renewable sources (zero value of parameter 10) is also unfavorable, although its consumption is low (parameter 11). The final test result (0.54) is moderately good and even slightly better than that obtained for the previously described titrimetric methods for calcium determination [49].

## 3. Materials and Methods

### 3.1. Materials

Carrageenan samples came from four suppliers: Sigma Aldrich, Burlington, MA, USA; Pol Aura, Morąg, Poland; Iguana Ltd., Peterborough, UK; and abcr GmbH, Karlsruhe, Germany (Table 3). Alcian Blue-tetrakis(methylpyridinium) chloride (AB) and poly(sodium 4-styrenesulfonate) (PSS) came from Sigma-Aldrich. The average molecular weight of the PSS used is approximately 70,000 g/mol. The purity of PSS is designated as 200 in the classification system used by Sigma-Aldrich, which means standard laboratory reagent for chemical analysis. A 35–38% hydrochloric acid solution was purchased from Chempur, Piekary Śląskie, Poland.

### 3.2. Methods

#### 3.2.1. Standardization of the AB Dye Solution

AB aqueous solutions of various concentrations were prepared by dissolving the appropriate amount in distilled water. They were then filtered through filter paper and stored in the dark. The actual concentration of the dye solution was determined against the 2.5 mM PSS solution. Suspensions of PSS-AB ionic associates were prepared by adding varying amounts of PSS stock solution to a fixed volume (5 mL) of AB solution, and then supplementing with distilled water to a final volume of 8 mL. The mixtures were mixed and left for 24 h for sedimentation. The number of reactive cationic groups in the dye was calculated from the amounts of PSS and dye in the mixture, which revealed deep sedimentation [36].

#### 3.2.2. Association of the AB Dye with Carrageenan

Carrageenan solutions at concentrations of 1 or 4 g/L were prepared by dissolving a weighed amount in distilled water at 70 °C. Tests were carried out on a series of solutions containing a constant dose (i.e., 5 mL) of dye and a variable dose of carrageenan. The concentrations of the dye solutions were in the range from 95.9 to 1150 mg/L. After adding a portion of the carrageenan solution, the mixtures were made up to the same volume with distilled water, mixed, and left for sedimentation. Two replicates were performed for each mixture.

#### 3.2.3. Measurement Methods

The moisture content of carrageenan samples was determined using a moisture analyzer MAX 210/WH (Radwag, Radom, Poland).

Spectral measurements were carried out using a Genesys 50 UV–vis spectrophotometer (Thermo Fisher Scientific, Waltham, MA, USA). The UV–vis spectrum was measured in a quartz cuvette with an optical path of 5 mm. Optical density at 600 nm was measured in disposable cuvettes with an optical path of 4 mm. Sedimentation of the carrageenan–dye associate was recorded at 600 nm using glass test tubes of 14 mm in diameter.

Microscopic images of the freshly precipitated precipitate were taken using a B-500 optical microscope (Optika, Ponteranica, Italy). ATR-FTIR spectra of the AB dye, carrageenans, and dried precipitates were recorded using an Alpha-P spectrometer (Bruker, Billerica, MA, USA) with a diamond window.

The reference determination of sulfate content in carrageenan samples was carried out using a two-step procedure. First, an exact sample mass of 50–80 mg was dissolved in 20 mL of 4N HCl and boiled for 4 h. This solution was cooled, filled to 200 mL, and the concentration of sulfate ions measured using the SulfaVer 4 reagent kit and a DR3900 spectrophotometer (Hach Company, Loveland, CO, USA).

## 4. Conclusions

The phenomenon of association of the Alcian Blue dye with anionic polymers has been used to determine sulfate groups in commercial carrageenans. It was shown that carrageenan can bind the AB dye and remove it from solution at a certain mass ratio of the two components. At this ratio, the ion associate that formed undergoes deep sedimentation. This is the equivalent point for the titration of the dye with carrageenan, which allows for the determination of the polymer’s anionic group content. The working AB solution was standardized using the previously described phenomenon of association of the Alcian Blue dye with the synthetic polymer poly(sodium styrene sulfonate). Tests carried out on carrageenan samples of various types from different manufacturers showed the usefulness of the proposed method. The proposed method was evaluated in terms of environmental friendliness. According to the AGREE test, the score obtained is 0.54. The method uses simple equipment such as scales and automatic pipettes and is easy to use. The proposed method avoids the use of concentrated acids or toxic organic solvents, as in the case of other methods [16,17,20]. The required reagent, Alcian Blue dye, is readily available and has moderate toxicity. The coagulation of ion associates takes only 15–30 min, which allows for a reliable determination of the critical carrageenan-to-dye ratio. No significant limitations of the AB coagulation method have been identified. All these advantages make the proposed method suitable for routine analysis in small industrial plants.

## Figures and Tables

**Table 1:** Main bands in FTIR spectra of AB dye, carrageenans, and ion associates.

Band, cm^−1^							Description	Refs.
AB Dye	Carrageenan			Ion Associate				
	κ-SA	ι-SA	λ-SA	AB + κ-SA	AB + ι-SA	AB + λ-SA		
	3300	3367	3291	3300	3315	3260	–OH	
3400–3100				3400–3100	3400–3100	3400–3100	N-substituted pyridinium	[43,44]
	1640	1640	1640	1640	1640	1639	–OH	
1610				1610	1610	1610	N-substituted pyridinium	[43,44]
1486				1486	1486	1486	N-substituted pyridinium	[43,44]
1390				1390	1390	1390	N-substituted pyridinium	[43,44]
	1347	1349	-	-	1349	-	H–C–H bending	[45,46]
	1230	1216	1227	-	1215	1211	ester sulfate	[19,20,21]
1151				1151	1151	1151	N-substituted pyridinium	[43,44]
1102				1102	1102	1102	N-substituted pyridinium	[43,44]
	1064	1066	1064	1064	1066	1064	ester sulfate	[19,20,21]
	1046	-	1040	1050	-	1040	C-O-C bonds	[20]
	917	928	929	922	925	926	C-O-C bonds	[20]
	845	846	849	840	839	832	ester sulfate	[19,20,21]
	-	803	804	-	806	804	–O–SO_3_ stretching vibrations	[47]
735				735	735	735	N-substituted pyridinium	[43,44]
	702	703	701	702	695	695	ester sulfate	[19,20,21]

**Table 2:** The assessment criteria according to the AGREE test.

No.	Principle	Value	Weight
1	Direct analytical techniques should be applied to avoid sample treatment	Off-line analysis (0.48)	2
2	Minimal sample size and minimal number of samples are goals	Max. 3 mL/sample (0.49)	1
3	In situ measurements should be performed	Off-line (0.00)	2
4	Integration of analytical processes and operations saves energy and reduces the use of reagents	2 or fewer (1.00)	3
5	Automated and miniaturized methods should be selected	Manual/non (0.50)	2
6	Derivatization should be avoided	No derivatization (1.00)	2
7	Generation of a large volume of analytical waste should be avoided and proper management of analytical waste should be provided	Max. 100 mL (0.08)	2
8	Multianalyte or multiparameter methods are preferred versus methods using one analyte at a time	1 analyte/run, 1 sample/h (0.05)	2
9	The use of energy should be minimized	Titration (1.00)	2
10	Reagents obtained from renewable sources should be preferred	None of the reagents are from bio-based sources (0.00)	2
11	Toxic reagents should be eliminated or replaced	0.05 g (0.80)	2
12	The safety of the operator should be increased	Corrosive (0.80)	2

**Table 3:** Carrageenan samples used in the study.

Type	Supplier ^*)^	Code	Moisture, %	Sulfate, mmol/g
kappa	Sigma Aldrich	κ-SA	10.3	2.07
kappa	Pol Aura	κ-PA	11.5	2.25
kappa	Iguana	κ-IG	5.61	0.68
iota	Sigma Aldrich	ι-SA	12.8	3.95
iota	Pol Aura	ι-PA	11.4	2.49
lambda	Sigma Aldrich	λ-SA	11.5	2.81
lambda	abcr GmbH	λ-abcr	13.3	4.26

^*)^ Detailed manufacturer data is provided in the text above the table.

## Data Availability

All data are contained within the article.

## Abbreviations

The following abbreviations are used in this manuscript:

AB	Alcian Blue dye
FTIR	Fourier-transform infrared spectroscopy
ι-SA	iota-carrageenan from Sigma Aldrich
ι-PA	iota-carrageenan from Pol Aura
κ-SA	kappa-carrageenan from Sigma Aldrich
κ-PA	kappa-carrageenan from Pol Aura
κ-IG	kappa-carrageenan from Iguana
λ-SA	lambda-carrageenan from Sigma Aldrich
λ-abcr	lambda-carrageenan from abcr GmbH
NMR	nuclear magnetic resonance spectroscopy
PSS	poly(styrene sulfonate)
